# Longitudinal trajectories of functional recovery after hip fracture

**DOI:** 10.1371/journal.pone.0283551

**Published:** 2023-03-29

**Authors:** Shams Dakhil, Ingvild Saltvedt, Jūratė Šaltytė Benth, Pernille Thingstad, Leiv Otto Watne, Torgeir Bruun Wyller, Jorunn L. Helbostad, Frede Frihagen, Lars Gunnar Johnsen, Kristin Taraldsen

**Affiliations:** 1 Oslo Delirium Research Group, Department of Geriatric Medicine, Oslo University Hospital, Oslo, Norway; 2 Institute of Clinical Medicine, University of Oslo, Oslo, Norway; 3 Department of Geriatric Medicine, St. Olav University Hospital, Trondheim, Norway; 4 Department of Neuromedicine and Movement Science, Norwegian University of Science and Technology (NTNU), Trondheim, Norway; 5 Institute of Clinical Medicine, Campus Ahus, University of Oslo, Oslo, Norway; 6 Health Services Research Unit, Akershus University Hospital, Lørenskog, Norway; 7 Department of Orthopaedic Surgery, Østfold Hospital Trust, Grålum, Norway; 8 Orthopedic Trauma Unit, Department of Orthopedic Surgery, St. Olav University Hospital, Trondheim, Norway; 9 Department of Rehabilitation Science and Health Technology, OsloMet, Oslo Metropolitan University, Oslo, Norway; Medical College of Wisconsin, UNITED STATES

## Abstract

**Background:**

There is limited evidence regarding predictors of functional trajectories after hip fracture. We aimed to identify groups with different trajectories of functional recovery the first year after hip fracture, and to determine predictors for belonging to such groups.

**Methods:**

This longitudinal study combined data from two large randomized controlled trials including patients with hip fracture. Participants were assessed at baseline, four and 12 months. We used the Nottingham Extended Activities of Daily Living (NEADL) as a measure of instrumental ADL (iADL) and Barthel Index for personal ADL (pADL). A growth mixture model was estimated to identify groups of patients following distinct trajectories of functioning. Baseline characteristics potentially predicting group-belonging were assessed by multiple nominal regression.

**Results:**

Among 726 participants (mean age 83.0; 74.7% women), we identified four groups of patients following distinct ADL trajectories. None of the groups regained their pre-fracture ADL. For one of the groups identified in both ADL outcomes, a steep decline in function was shown the first four months after surgery, and none of the groups showed functional recovery between four and 12 months after surgery.

**Conclusions:**

No groups regained their pre-fracture ADL. Some of the patients with relatively high pre-fracture function, had a steep ADL decline. For this group there is a potential for recovery, but more knowledge and research is needed in this group. These findings could be useful in uncovering groups of patients with different functioning after a hip fracture, and aid in discharge planning.

## Introduction

With an ageing population, the number of hip fractures is expected to rise, even though the trends suggest a stabilization in age-adjusted rates [1, 2]. This condition in older adult patients is often associated with adverse outcomes, such as increased mortality and morbidity [3], increased dependency in Activities of Daily Living (ADL) [4–6], reduced mobility [7], and a negative effect on cognitive function [8, 9]. In addition to the individual negative outcomes, the increased dependency will also place a high demand on health care systems in the years to come. Thus, hip fractures constitute a major socioeconomic and public health burden worldwide [10, 11], and it is of value to investigate the best method for treating these patients.

Older adults suffering from a hip fracture are often characterized as frail, with multiple comorbidities and polypharmacy [12]. Many also have cognitive impairment or dementia [13], that increases the risk of falls and fractures [13], as well as for negative outcomes such as delirium [12], longer hospitalizations, a higher risk of death and nursing home admissions, and reduced functional recovery [14, 15]. Closely related is also a reduction in functional levels, both pre- and post-fracture [16]. Needless to say, this is a large and complex group of older adult patients, requiring many resources. Post-fracture recovery may therefore be impacted by many factors, and studying these factors to increase our knowledge about post-fracture recovery can aid in better treatment and discharge planning for this group of older adults.

A significant amount of hip fracture patients do not regain their pre-fracture function [6]. Having suffered a hip fracture is associated with more disability in personal Activities of Daily Living (pADL) and in instrumental ADL (iADL) [3], which could lead to an overall loss of confidence and independency [5]. Furthermore, a decline in ADL is subsequently associated with negative outcomes, such as reduced quality of life and increased nursing home admissions [17]. Thus, it is important to study ADL and the recovery of ADL after a hip fracture, to understand the mechanisms behind the decline. This knowledge could, in turn, lead to better and more personalized rehabilitation after a hip fracture.

Many older adults have declining functions already before the hip fracture occurs [18], which may be a contributing factor both to the hip fracture itself and the overall decline seen after the fracture. Due to the major impact ADL has on the lives of the older adults and the society, investigating ADL and its recovery in older adults suffering a hip fracture may therefore be of value.

Several studies aiming at identifying groups following distinct trajectories of function after hip fracture have been published in recent years [19–21]. One such study aimed to identify different groups following distinct trajectories of recovery, and found that frail hip fracture patients were more likely to belong to a trajectory with worse recovery compared to non-frail hip fracture patients, and that poor recovery was associated with dementia [22]. Identifying such groups and patient characteristics associated with these, may be useful in determining rehabilitation potential and targeting treatment to each patient. In turn, this may lead to a more individualized and cost-effective organization of hip fracture care and rehabilitation, and better function and health related quality of life among hip fracture patients [23]. By using a large and heterogeneous data material, we aimed to investigate whether there were homogenous groups of patients following different trajectories of recovery of physical function the first year after hospital discharge related to hip fracture, and to determine the most important predictors for belonging to such groups. We hypothesized that trajectories of functioning during the first year after the fracture vary depending on patient characteristics before the fracture.

## Materials and methods

### Design

This is a longitudinal study based on data from two randomized clinical trials conducted in Norway [12, 23]; The Oslo Orthogeriatric Trial (n = 329, inclusion between 2009–2012) and the Trondheim Hip Fracture Trial (n = 397, inclusion between 2008–2010). Both studies aimed to evaluate the effect of orthogeriatric care and were planned with similar design for future pooling of the data [24, 25]. The population did, however, slightly differ between the two studies. The Trondheim study only included home-dwelling patients, 70 years or older, who were able to walk at least 10 meters before the hip fracture. The Oslo study included all low-energy hip fracture patients at all ages, independent of place of residence. Both studies excluded patients who were moribund at admission or had suffered a hip fracture due to high-energy trauma. In both studies, participants received comprehensive geriatric care (CGC) in a geriatric ward or usual care in an orthopedic ward (OC) during the hospital stay, full details on the intervention are described in their study protocols [24, 25]. Patients were followed one year after surgery, with assessments at baseline, four and 12 months. The Oslo Orthogeriatric Trial found no effect of the intervention on cognitive function (primary endpoint), however there was an effect on mobility four months after surgery for home-dwelling patients (pre-planned subgroup analysis) [12]. For the Trondheim Hip Fracture Trial, better mobility and iADL was found for the intervention group four months after surgery, and the intervention was beneficial for most secondary outcomes, as well as being cost effective up to one year after surgery [23].

### Sample and setting

In the present study, we used the pooled data from the two studies, yielding a database with 726 participants.

### Measurements

#### Descriptive measures

Baseline characteristics included randomization (CGC vs OC), sex (male/female), age (years), type of fracture (extracapsular vs intracapsular) and preoperative waiting time (hours). The preoperative physical health was assessed by the American Society of Anesthesiologists (ASA) score, a classification system using four categories of physical status, which were dichotomized (1 or 2 vs 3 or 4) in this study [26]. In addition, cognitive function at baseline was assessed using the Clinical Dementia Rating Scale (CDR) [27], which is a global rating scale, where current functioning in six domains is rated based on changes in cognitive function from previous usual levels. By adding the scores for each item, the CDR sum of boxes, ranging from 0 to 18, is achieved; a low sum score indicating little or no cognitive impairment [28].

#### Outcomes

We included two functional outcomes; instrumental and personal ADL. We used the Nottingham Extended ADL Scale (NEADL) to measure **iADL** [29]. NEADL is a 22 items scale with scores ranging from 0 to 66, where a higher score indicates better iADL [29]. We used the Barthel ADL Index (BADL) to measure **pADL** [30]. BADL is a 10-item scale with scores ranging from 0 to 20, where a higher BADL score suggests higher independency in undertaking pADL [30]. Both outcomes were collected at baseline, four and 12 months postoperatively, where the baseline value represents patients’ pre-fracture function and obtained by proxy interview asking for function 14 days before the fracture, and the value at both follow-ups were obtained by proxy interview and face-to-face evaluations.

### Statistical methods

Participant characteristics were described as means and standard deviations (SDs) or frequencies and percentages.

Growth mixture models [31] were used to identify possible homogeneous groups of participants following distinct trajectories in NEADL and BADL. This approach is suitable for identifying groups of patients based on their individual profiles by using several statistical criteria. To determine the number of groups that best cover the heterogeneity in participants’ profiles, Bayes Information Criterion, where a smaller value means a better model, was applied. In addition, an average within-group probability of at least 0.80, reasonable group sizes, and non-overlapping 95% confidence intervals (CIs) of the group trajectories were required. Patients completing at least baseline test were included in the analyses.

Patient characteristics within different groups were presented as frequencies and percentages or means and SDs. Multiple nominal regression models were used to assess which baseline characteristics (sex, age, type of fracture, preoperative waiting time, ASA score and CDR) were associated with group-belonging. In all models, the largest group was used as reference. As the data were collected from different hospitals, a cluster effect might be present. The cluster effect was assessed by intra-class correlation coefficient. If present, it was adjusted for by including random effects for hospital into the nominal regression model. The variable for care models, CGC or OC, was treated as control variable in our analysis. The analysis included patients with no missing values on considered characteristics. The results were presented as odds ratios (ORs) with corresponding 95% CIs and p-values.

All tests were two-sided and results with p-values < 0.05 were considered statistically significant. The analyses were performed by using SPSS v26, SAS v9.4, and STATA v14.

### Ethical considerations

The Oslo Orthogeriatric Trial was registered with ClinicalTrials.gov (NCT01009268), and approved by the Regional Committee for Ethics in Medical Research in South East of Norway (REK 2009/450). The Trondheim Hip Fracture Trial was registered with ClinicalTrials.gov (NCT00667914), and approved by the Regional Committee for Ethics in Medical Research in Central Norway (REK4.2008.335). The Regional Committee for Ethics in Medical Research in South East of Norway and the Data Protection Officer at both hospitals approved merging of data from the two separate trials.

Both studies were conducted in accordance with the Declaration of Helsinki. The patients or a proxy gave informed written consent to be included in the study before participation in both trials.

## Results

We included 726 participants (mean age 83.0 (7.7) years, 74.7% women, 60.7% intracapsular fracture). Out of the 726 participants, 361 were randomized to CGC and 365 were randomized to OC, with no between-group differences in baseline characteristics [32]. Participants’ baseline characteristics are presented in Table 1.

**Table 1:** Baseline characteristics.

Subjects, n	726
Age, mean (SD)	83.0 (7.7)
Sex, female (%)	542 (74.7)
Type of fracture, intracapsular (%)	441 (60.7)
Living in a nursing home at admission^a^ (%)	102 (14.0)
NEADL^b^ (0–66), mean (SD)	37.3 (20.2)
BADL^c^ (0–20)	17.3 (3.6)
CDR sum of boxes^d^ (0–18), mean (SD)	4.2 (5.4)
Preop. waiting time^e^^,^^f^ (hours), mean (SD)	29.9 (23.3)
ASA^g^ score 3 or more (%)	415 (59.2)
Patients included from Trondheim (%)	397 (54.7)
Randomization to intervention, CGC (%)	361 (49.7)

*SD* Standard Deviation. *NEADL* Nottingham Extended Activities of Daily Living. *BADL* Barthel Index for Activities of Daily Living. *CDR* Clinical Dementia rating Scale. *Preop*. *waiting time* Preoperative waiting time. *ASA* American Society of Anaesthesiologists Physical Status Classification system. *CGC* Comprehensive Geriatric Care.

^a^ Patients admitted from a nursing home was excluded in Trondheim.

^b^ Baseline NEADL was based on pre-fracture function, and was obtained by proxy-interview. It was missing from 21 patients.

^c^ Baseline BADL was based on pre-fracture function, and was obtained by proxy-interview. It was missing from 16 patients.

^d^ Baseline CDR sum of boxes was obtained during hospital and was, in part based on proxy-interview. It was missing in 52 patients.

^e^ Outlier, 235h.

^f^ Information about preoperative waiting time was missing in 9 patients.

^g^ ASA score was missing in 25 patients.

**Fig 1:**
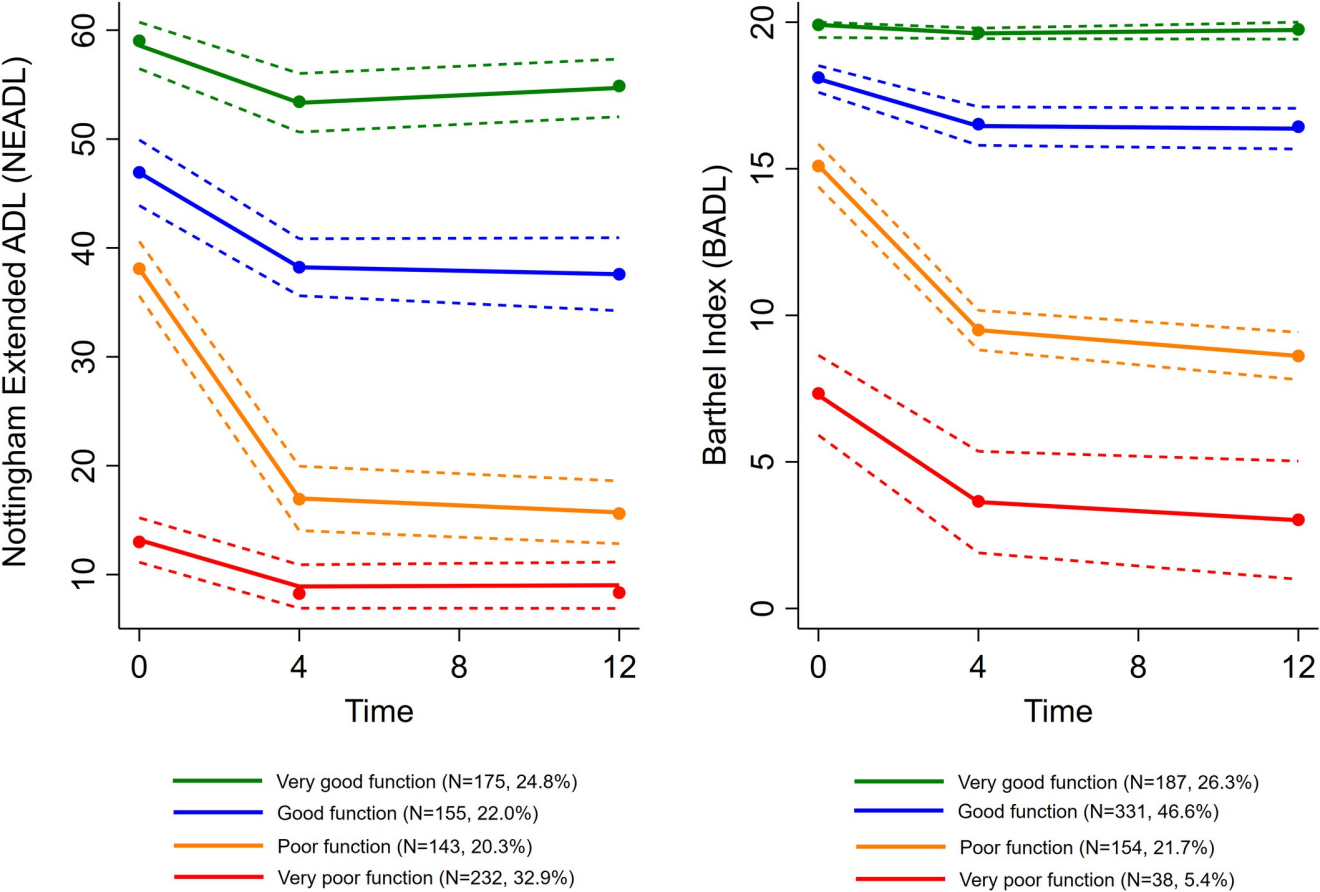
Growth mixture models for instrumental activities of Daily Living (NEADL) and personal activities of Daily Living (BADL) with corresponding confidence intervals.

Four different groups of patients following distinct trajectories for each of the two ADL variables were identified, see Fig 1. For iADL, the two groups, *‘Very good function’* (n = 175, 24.8%) and *‘Good function’* (n = 155, 22.0%) comprised roughly half of the patients. The *‘Poor function’* (n = 143, 20.3%) group showed relatively high baseline iADL (mean 31.5), but declined steeply the first four months after the fracture (mean 15.7 at last assessment). The *‘Very poor function’* (n = 232, 32.9%) group showed low pre-fracture iADL (mean 11.8) and were relatively stable. For pADL, two groups maintained relatively good function, the *‘Very good function’* (n = 187, 26.3%, mean 19.9 at baseline) and *‘Good function’* (n = 331, 46.6%, mean values 17.6, 16.5 and 16.4 at each assessment, respectively) groups, whereas two groups had a steep decline in pADL the first four months after hip fracture; ‘*Poor function’* (n = 154, 21.7%, mean values 13.4, 9.5 and 8.6 at each assessment, respectively) and *‘Very poor function’* (n = 38, 5.4%, mean values 6.1, 3.6 and 3.0 at each assessment, respectively). Average group probabilities were all above 0.8 and 95% CIs non-overlapping, implying homogeneous groups. For both iADL and pADL, all trajectories were non-linear and declined significantly over 12 months (all p’s <0.001 and <0.01, respectively). See Table 2 for details.

**Table 2:** Results of the growth mixture model.

	Mean (SD) at baseline	Linear component		Quadratic component	
		Regression coefficient (SE)	p-value	Regression coefficient (SE)	p-value
**Instrumental Activities of Daily Living (NEADL)**					
Very poor function (G1) (n = 232, 32.9%)	13.01 (7.9)	-1.61 (0.35)	**<0.001**	0.10 (0.03)	**<0.001**
Poor function (G2) (n = 143, 20.3%)	38.3 (9.4)	-7.00 (0.60)	**<0.001**	0.43 (0.04)	**<0.001**
Good function (G3) (n = 155, 22.0%)	47.4 (9.7)	-2.88 (0.44)	**<0.001**	0.18 (0.03)	**<0.001**
Very good function (G4) (n = 175, 24.8%)	59.7 (5.6)	-2.11 (0.38)	**<0.001**	0.14 (0.03)	**<0.001**
**Personal Activities of Daily Living (BADL)**					
Very poor function (G1) (n = 38, 5.4%)	7.0 (3.6)	-1.23 (0.29)	**<0.001**	0.07 (0.02)	**0.002**
Poor function (G2) (n = 154, 21.7%)	14.7 (2.7)	-1.85 (0.14)	**<0.001**	0.11 (0.01)	**<0.001**
Good function (G3) (N = 331, 46.6%)	18.2 (1.7)	-0.66 (0.09)	**<0.001**	0.04 (0.01)	**<0.001**
Very good function (G4) (n = 187, 26.3%)	20.0 (0.2)	-0.69 (0.22)	**0.002**	0.05 (0.02)	**0.002**

Growth mixture models for instrumental Activities of Daily and personal Activities of Daily Living. *SD* Standard deviation. *SE* Standard error. *NEADL* Nottingham Extended Activities of Daily Living. *BADL* Barthel Index for Activities of Daily Living.

Participants’ characteristics stratified by the trajectory groups are presented in Table 3. For both iADL and pADL, mean age was lowest and no participant was admitted from a nursing home in the *‘Very good function’* groups. Furthermore, for both the *‘Very good function’* and the *‘Good function’* groups for both iADL and pADL, lower ASA score and an intracapsular fracture were more common.

**Table 3:** Characteristics of patients according to trajectories.

Variable	Subjects *n (%)*	Age *years (SD)*	Female Sex *n (%)*	Nursing home *n (%)*	ASA score 1+2 *n (%)*	ASA score 3+ *n (%)*	Type of fracture, Intracapsular *n (%)*
Overall	726 (100)						
Instrumental Activities of Daily Living (NEADL)							
Very poor function (G1)	232 (32.9)	85.2 (6.6)	165 (71.1)	97 (41.8)	57 (25.9)	163 (74.1)	141 (60.8)
Poor function (G2)	143 (20.3)	85.1 (7.0)	107 (74.8)	2 (1.4)	49 (35.3)	90 (64.7)	76 (53.1)
Good function (G3)	155 (22.0)	82.2 (7.4)	121 (78.1)	1 (0.6)	71 (46.4)	82 (53.6)	91 (58.7)
Very good function (G4)	175 (24.8)	79.0 (8.3)	131 (74.9)	0	100 (59.9)	67 (40.1)	119 (68.0)
Personal Activities of Daily Living (BADL)							
Very poor function (G1)	38 (5.4)	82.9 (8.1)	28 (73.7)	28 (73.7)	6 (17.6)	28 (82.4)	27 (71.1)
Poor function (G2)	154 (21.7)	85.7 (6.0)	108 (70.1)	52 (33.8)	31 (20.5)	120 (79.5)	81 (52.6)
Good function (G3)	331 (46.6)	83.7 (7.4)	267 (80.7)	20 (6.0)	137 (42.9)	182 (57.1)	195 (58.9)
Very good function (G4)	187 (26.3)	79.6 (8.2)	126 (67.4)	0	106 (58.9)	74 (41.1)	127 (67.9)

Characteristics of patients according to trajectories.

*SD* Standard deviation. *ASA score* American Society of Anaesthesiologists Physical Status Classification system. *iADL* Instrumental Activities of Daily Living. *NEADL* Nottingham Extended Activities of Daily Living. *pADL* Personal Activities of Daily Living. *BADL* Barthel Index for Activities of Daily Living

In the *‘Very poor function’* groups for both iADL and pADL, being admitted from a nursing home, a high ASA score and an intracapsular fracture were more common.

For both the *‘Poor function’* groups, higher age and higher ASA score were more common.

Table 4 presents the results from the multiple nominal regression models. For iADL, higher age was associated with lower odds of being in the *´Good function’* (OR 0.94, p = 0.003) and ´*Very good function´* (OR 0.89, p<0.001) groups, and higher CDR sum was associated with higher odds of being in the ´*Very poor function´* group (all p’s<0.001). Furthermore, ASA score of 1 or 2 compared to a higher ASA score, was associated with higher odds of belonging to the ´*Good function´* (OR 1.84, p = 0.048) and *‘Very good function´* (OR 3.28, p<0.001) groups. For pADL, men compared to women had higher odds of belonging to the *‘Very good function’* group (OR 3.29, p<0.001). Increasing age was associated with lower odds of belonging to the *‘Very poor function’* (OR 0.93, p = 0.028) and *‘Very good function’* (OR 0.94, p<0.001) groups, and increasing CDR sum was associated with higher odds of belonging to the *‘Very poor function’* (OR 1.57, p<0.001) group. Moreover, lower ASA score was associated with higher odds of belonging to the *‘Very good function’* group (OR 2.17, p = 0.001), and having suffered an extracapsular hip fracture were associated with lower odds of belonging to the *‘Very good function’* (OR 0.49, p = 0.002) group.

**Table 4:** Results of the multiple nominal regression model.

Covariate	Multiple model for iADL (NEADL), N = 641		Multiple model for pADL (BADL), N = 645	
	OR (95% CI)	p-value	OR (95% CI)	p-value
Randomization				
Orthopedic (OC)	1		1	
Geriatric (CGC)				
*Very poor function (G1)*	1		1.27 (0.50; 3.27)	0.615
*Poor function (G2)*	0.62 (0.37; 1.05)	0.077	0.83 (0.51; 1.35)	0.446
*Good function (G3)*	0.90 (0.51; 1.59)	0.713	1	
*Very good function (G4)*	1.12 (0.60; 2.09)	0.729	0.89 (0.58; 1.37)	0.605
Sex				
Male				
*Very poor function (G1)*	1		0.63 (0.21; 1.92)	0.418
*Poor function (G2)*	0.94 (0.51; 1.73)	0.846	1.31 (0.74; 2.31)	0.354
*Good function (G3)*	0.87 (0.44; 1.70)	0.681	1	
*Very good function (G4)*	1.15 (0.55; 2.39)	0.718	3.29 (1.97; 5.52)	<0.001
Female	1		1	
Age				
*Very poor function (G1)*	1		0.93 (0.86; 0.99)	0.028
*Poor function (G2)*	1.00 (0.96; 1.04)	0.877	1.03 (0.99; 1.07)	0.136
*Good function (G3)*	0.94 (0.90; 0.98)	0.003	1	
*Very good function (G4)*	0.89 (0.85; 0.94)	<0.001	0.94 (0.92; 0.97)	<0.001
Type of fracture				
Extracapsular				
*Very poor function (G1)*	1		0.73 (0.27; 1.98)	0.539
*Poor function (G2)*	1.33 (0.78; 2.26)	0.295	1.51 (0.93; 2.47)	0.100
*Good function (G3)*	0.97 (0.54; 1.72)	0.905	1	
*Very good function (G4)*	0.59 (0.31; 1.12)	0.107	0.49 (0.31; 0.77)	0.002
Intracapsular	1		1	
Preop. waiting time				
*Very poor function (G1)*	1		1.00 (0.99; 1.02)	0.641
*Poor function (G2)*	1.00 (0.99; 1.01)	0.913	1.00 (0.99; 1.01)	0.997
*Good function (G3)*	1.01 (0.99;	0.236	1	
*Very good function (G4)*	1.02)	0.547	1.00 (0.99; 1.01)	0.727
ASA score	1.01 (1.00;			
1 or 2	1.02)			
*Very poor function (G1)*			0.27 (0.08; 0.90)	0.034
	1			
*Poor function (G2)*	1.24 (0.71; 2.18)	0.457	0.42 (0.24; 0.73)	0.002
*Good function (G3)*	1.84 (1.01; 3.35)	0.048	1	
*Very good function (G4)*	3.28 (1.70; 6.32)	<0.001	2.17 (1.40; 3.38)	0.001
3 or more	1		1	
CDR sum of boxes				
*Very poor function (G1)*	1		1.57 (1.39; 1.77)	<0.001
*Poor function (G2)*	0.76 (0.71; 0.81)	<0.001	1.29 (1.23; 1.39)	<0.001
*Good function (G3)*	0.61 (0.55; 0.67)	<0.001	1	
*Very good function (G4)*	0.35 (0.28; 0.44)	<0.001	0.66 (0.58; 0.75)	<0.001

Multiple nominal regression model with CGC or OC as control variable. The analysis included patients with no missing values on considered characteristics. OR = 1 indicates odds ratios for the reference (largest) group.

*iADL* Instrumental Activities of Daily Living. *NEADL* Nottingham Extended Activities of Daily Living. *pADL* Personal Activities of Daily Living. *BADL* Barthel Index for Activities of Daily Living. *OR* Odds ratio. *CI* Confidence interval. *OC* Orthopedic care. *CGC* Comprehensive Geriatric Care. *Preop*. *waiting time* Preoperative waiting time. *ASA score* American Society of Anaesthesiologists Physical Status Classification system. *CDR sum of boxes* Clinical Dementia rating Scale sum of boxes.

For iADL and pADL, 316 participants (44.6%) belonged to the same trajectory group in both outcomes (for example 38 participants belong to the *‘Very poor function’* group for iADL and the corresponding *‘Very poor function’* group for pADL). The cross-table presenting agreement between group-belonging (see Table 5) was followed by a kappa of 0.46 (CI: 0.42–0.50), which is consistent with moderate agreement across the groups.

**Table 5:** Crosstabulation between iADL and pADL groups.

Groups	Personal Activities of Daily Living (BADL) groups					Total
		G1	G2	G3	G4	
Instrumental Activities of Daily Living (NEADL) groups	G1, count (% of total)	38 (5.4)	120 (17.0)	74 (10.5)	0 (0)	232 (33.0)
	G2, count (% of total)	0 (0)	33 (4.7)	101 (14.3)	9 (1.3)	143 (20.3)
	G3, count (% of total)	0 (0)	0 (0)	111 (15.8)	44 (6.3)	155 (22.0)
	G4, count (% of total)	0 (0)	0 (0)	40 (5.7)	134 (19.0)	174 (24.7)
Total, count (% of total)		38 (5.4)	153 (21.7)	326 (46.3)	187 (26.6)	704 (100)

Crosstabulation presenting agreement between group-belonging, which is consistent with moderate agreement across groups (kappa 0.46).

*iADL* Instrumental Activities of Daily Living. *pADL* Personal Activities of Daily Living. *NEADL* Nottingham Extended Activities of Daily Living. *BADL* Barthel Index for Activities of Daily Living.

*G1* Very poor function. *G2* Poor function. *G3* Good function. *G4* Very good function.

## Discussion

In this longitudinal cohort of 726 older adults we studied functional decline one year after hip fracture. The statistical analyses identified four groups following distinct trajectories for both iADL and pADL. For both iADL and pADL, most trajectories did not regain their pre-fracture ADL levels. Overall, younger age, an ASA score of 1 or 2, and lower CDR score were all associated with belonging to groups with higher ADL and better trajectories. We also identified a group of patients for both iADL and pADL with relatively good pre-fracture function, but with a steep decline in ADL function the first four months after the fracture. This decline remained one year after fracture.

As a difference higher than 2.4 points in NEADL is considered clinically relevant [33], and a one-point difference in BADL distinguishes being independent or dependent in certain items of pADL (such as for walking, feeding and toilet use), we believe all groups show a clinically relevant decline in ADL the first year after a hip fracture. Due to the higher complexity of iADL tasks, it is not unusual that patients first experience deterioration iADL and then in pADL, which might explain the kappa agreement of 46% in our analysis.

For all iADL and pADL trajectories, the functional decline was steepest the first four months after the fracture, with no functional recovery between four and 12 months. Whether it is because the participants had reached their maximum rehabilitation potential, or because the rehabilitation offered concluded prematurely, remains to be explored [34]. Nevertheless, these trajectories may represent different groups of patients with different rehabilitation needs, and a need for more personalized rehabilitation, especially in early phases when discharge are planned and during the first months after the hip fracture.

The large and steep decline in ADL in the group of patients following the *‘Poor function*’ trajectory of both iADL and pADL may represent a potential for improved acute care and rehabilitation, especially because of the relatively high pre-fracture ADL status of these participants. The group of patients following these trajectories experienced a gross decline in ADL the first four months after a hip fracture, which persisted over the following year. These groups were characterized by older patients with higher ASA scores.

ASA score is a measure of preoperative function, and a reflection of the patients’ preoperative clinical status, comorbidities and physical fitness. Higher ASA score is associated with higher mortality [35], longer hospitalization [36] and more hospital readmissions [37], and is an important prognostic factor. Identifying patients belonging to these groups might be clinically relevant, since correcting for comorbidities and optimizing treatment, as well as intensified rehabilitation for such patients could be of importance to avoid the large decline in ADL. Furthermore, the groups of patients following the *‘Poor function’* trajectory for both iADL and pADL were mostly home-dwellers (1.4% and 33.8% admitted from a nursing home, respectively), see Table 3. Theoretically, these patients should be less frail, but when admitted to the hospital after a hip fracture, they have high ASA scores reflecting frailty or acute disease before or during the hip fracture. The high ASA score in this group could be a contributing factor to their steep decline in ADL–either by reflecting a disease that contributes to the fall and fracture, or by reflecting an innate frailty that subsequently result in worse ADL recovery. The mechanisms behind this are not yet known. Future research on this group of patients is important to increase the evidence regarding adequate acute treatment and rehabilitation that can be offered in this group.

In our sample, over half of the older adults with hip fracture were in the two lower groups of iADL, with approximately 30% in the lowest group in which iADL was already poor before the fracture. These groups stand out, probably illustrating that for some older adults their already low pre-fracture iADL could be a contributing factor to the fall, subsequent hip fracture and overall decline in function postoperatively. This is in alignment with literature finding that pre-fracture function is an important factor for post-fracture functional recovery [38–42].

The major strengths of this study are the relatively high number of participants that are followed for one year after hip fracture, and the comprehensive and systematic collection of clinically relevant outcomes. The study participants were representative of older adults with hip fracture, with the Oslo study including hip fracture patients regardless of living conditions prior to the fracture and the Trondheim study including home-dwelling hip fracture patients above 70 years. Our results indicate that growth mixture modelling can be a useful tool in identifying homogeneous groups of patients following distinct trajectories of ADL after hip fracture. The limitations of this study include that some outcomes collected at baseline by proxy interview could be biased by the knowledge of the recent hip fracture. We also acknowledge that the Trondheim cohort did not include nursing home residents, thus explaining the lower proportion of nursing home residents in our material.

In summary, we identified four groups of older adults with hip fracture that followed distinct trajectories of iADL and pADL the first year after the fracture. Younger age, an ASA score of 1 or 2, and better cognitive function at baseline were all associated with belonging to a group with better ADL. For all groups there was no functional recovery between four and 12 months after the fracture, and no group showed recovery to pre-fracture functional levels. We also identified a group with relatively high ADL before the fracture, followed by a steep decline afterwards. This group is of particular clinical interest since it may impose a significant potential for rehabilitation. Future studies should explore how to target treatment for groups of older adults with steep declines in functioning after a hip fracture. Our findings could potentially be useful for the quality, efficacy and type of care hip fracture patients should be offered, promoting construction of clinical profiles to aid in more individualized rehabilitation and discharge planning.
